# A Novel SALL4/OCT4 Transcriptional Feedback Network for Pluripotency of Embryonic Stem Cells

**DOI:** 10.1371/journal.pone.0010766

**Published:** 2010-05-21

**Authors:** Jianchang Yang, Chong Gao, Li Chai, Yupo Ma

**Affiliations:** 1 Division of Laboratory Medicine, Nevada Cancer Institute, Las Vegas, Nevada, United States of America; 2 The Department of Pathology, Brigham and Women's Hospital, Harvard Medical School, Boston, Massachusetts, United States of America; Brunel University, United Kingdom

## Abstract

**Background:**

SALL4 is a member of the SALL gene family that encodes a group of putative developmental transcription factors. Murine Sall4 plays a critical role in maintaining embryonic stem cell (ES cell) pluripotency and self-renewal. We have shown that Sall4 activates Oct4 and is a master regulator in murine ES cells. Other SALL gene members, especially Sall1 and Sall3 are expressed in both murine and human ES cells, and deletions of these two genes in mice lead to perinatal death due to developmental defects. To date, little is known about the molecular mechanisms controlling the regulation of expressions of SALL4 or other SALL gene family members.

**Methodology/Principal Findings:**

This report describes a novel SALL4/OCT4 regulator feedback loop in ES cells in balancing the proper expression dosage of SALL4 and OCT4 for the maintenance of ESC stem cell properties. While we have observed that a positive feedback relationship is present between SALL4 and OCT4, the strong self-repression of SALL4 seems to be the “break” for this loop. In addition, we have shown that SALL4 can repress the promoters of other SALL family members, such as SALL1 and SALL3, which competes with the activation of these two genes by OCT4.

**Conclusions/Significance:**

Our findings, when taken together, indicate that SALL4 is a master regulator that controls its own expression and the expression of OCT4. SALL4 and OCT4 work antagonistically to balance the expressions of other SALL gene family members. This novel SALL4/OCT4 transcription regulation feedback loop should provide more insight into the mechanism of governing the “stemness” of ES cells.

## Introduction

The SALL gene family (also called Hsal), comprised of SALL1, SALL2, SALL3, and SALL4, was originally cloned based on a DNA sequence homology to the Drosophila gene sal. In humans, SALL1 is mutated in patients with Townes-Brockes Syndrome (TBS), with features that include renal, limb, anal, and ear malformations [1], [2]. Sall1 null mutant mice die perinatally because of severe kidney dysgenesis or agenesis [3]. No human congenital malformation has been associated with SALL2 so far. SALL3 is mapped to chromosome 18q23, and it has been suggested that this isoform is involved in the phenotype of patients with 18q deletion syndrome, which is characterized by developmental delay, hypotonia, growth retardation, midface hypoplasia, hearing loss, and tapered fingers [4]. SALL3 null mice exhibit plate deficiency, abnormalities in cranial nerves, and perinatal lethality [5]. In human, SALL4 is mutated in patients with Duane Radial Ray Syndrome (DRRS, OMIM#126800) (also known as Duane Anomaly with Radial Ray abnormalities and Deafness syndrome or Okihiro syndrome) and Acro-renal-ocular syndrome [6], [7]. DRRS is an autosomal dominant disorder with the combination of Duane anomaly, radial ray abnormalities, and deafness. The clinical presentation of DR syndrome is highly variable. In addition to strabismus and limb malformation, these patients can have hearing defects, renal malformation, facial asymmetry and cardiac defects [8]. SALL4 mutations also result in a range of overlapping phenotypes, including Holt-Oram and Acro-renal-ocular syndrome, and IVIC syndrome [9], [10].

Parallel to its important role in development, the SALL gene family has been found to be expressed in human and murine ES cells and during early developments. SALL4 is expressed in the 2-cell stage of the embryo, similar to OCT4, while expression of SOX2 and NANOG begins in the blastocystic stage of embryonic development[11]–[13]. Our group and others have shown that murine Sall4 plays a vital role in maintaining ES cell pluripotency and in governing decisions affecting the fate of ES cells through transcriptional modulation of Oct4 and Nanog [11], [14]–[16], [13]. We and others have also shown that SALL4 can activate OCT4 and interact with Nanog [15]–[17], and the SALL4/OCT4/Nanog transcriptional core network is essential for the maintenance of “stemness” of ES cells [18]–[20].

Given its important function in ESC, we sought to investigate the transcriptional regulation of SALL4 in ES cells. We have identified that there are two human SALL4 isoforms (SALL4A and SALL4B) [21]. Here we show that both isoforms can activate the expression of OCT4 but suppress those of SALL1 and SALL3. In addition, we have observed that OCT4 can activate the transcription of SALL4, SALL1 and SALL3, suggesting that there is a positive transcription feedback loop between SALL gene family members and OCT4. While SALL1 had no effect on SALL4 promoter, surprisingly, SALL4 showed strong self-repression. Both SALL4 isoforms can repress its own promoter in a dose- dependent manner, and the activation of SALL4 by OCT4 is affected by the level of SALL4 expression. Our findings, when taken together, indicate that SALL4 expression is tightly regulated by self-repression and a positive feedback from OCT4. This novel SALL4/OCT4 transcription regulation feedback loop should provide more insight into the mechanism of governing the “stemness” of ES cells.

## Materials and Methods

### cDNA Cloning

We performed a tBLASTn search of the GenBank database (http://www.ncbi.nlm.nih.gov//) to identify mouse expressed sequence taqs (ESTs) with significant homology to human SALL4. ESTs highly homologous to the 5′ or 3′ noncoding regions of SALL4 were selected to design primers to amplify SALL4 cDNAs. The primers used were: 5′ primer, 5′-ATG TCG AGG CGC AAG CAG GCG AAG C-3′, and 3′ primer, 5′-TTA GCT GAC GGC AAT CTT ATT TTC C-3′. The entire coding regions of SALL4A and SALL4B were amplified from a mouse brain marathon- ready cDNA library (BD Biosciences Clontech, Palo Alto, CA), The amplified PCR products were cloned into a pcDNA3 vector (Invitrogen Corp., Carlsbad, CA), and the nucleotide sequences were determined by DNA sequencing.

### Cell culture and transfection

All cell cultures were maintained at 37°C with 5% CO2. The murine fibroblast cell lines NIH-3T3, monkey kidney cell lines COS-7 and the human embryonal kidney cell line HEK-293 (all from ATCC, Manassas, VA, USA)) were cultured in Dulbecco modified Eagle medium (DMEM) supplemented with 10% heat-inactivated FBS (fetal bovine serum) and penicillin/streptomycin (P/S). Transfection of plasmids into cultured cells was performed using Lipofectamine 2000 (Invitrogen, Carlsbad, CA) according to manufacturer's recommendations. Human ES cells H9 (Wicell, WI) and mouse W4 ES cells (kindly provided by the Gene Targeting Core Facility, University of Iowa) were cultured, either with feeders or in feeder-free conditions, as described previously [22], [13].

### Antibody generation

SALL4 antibody is generated as previously described [21].

### ChIP assay

HEK-293 cells (l×10^6^ cells/well in 6-well plates, with or without transient transfection, were processed using a ChIP Assay Kit (Upstate, Charlottesville, VA) following the manufacture's protocol. Briefly, cells were cross-linked by adding formaldehyde (27 µ1 of 37% formaldehyde/ml) and incubated for 10 min. Then, chromatin was sonicated to an average size of approximately 500 bp and immuno-precipitated with SALL4 antibodies, preimmune serum, or anti-HA (hemagglutination) antibody. Histone-DNA crosslinks were reversed by heating at 65°C followed by digestion with proteinase K (Invitrogen, Carlsbad, CA). DNA was recovered by using a PCR purification kit (Qiagen, Valcncia, CA) and then used for PCR or QRT- PCR (quantitative real time polymerase chain reaction).

### ChIP-chip Assay and Quantitative Real-time PCR (Q-PCR)

A complete protocol was provided by NimbleGen Systems Inc (Madison, WI). In brief, cells were grown, cross-linked with formaldehyde and sheared by sonication. The anti-SALL4 antibody and rabbit serum were used for chromatin immunoprecipitation (ChIP). ChIP-purified DNA was blunt-ended, ligated to linkers and subjected to low- cycle PCR amplification. Promoter tiling arrays (RefSeq array) were produced by NimbleGen. The RefSeq mouse promoter array design is a single array containing 2.7 kb of each promoter region (from build MM5). The promoter region is covered by 50–75 mer probes at roughly 100 bp spacing, depending on the sequence composition of the region. The arrays were hybridized, and the data were extracted according to NimbleGen standard procedures. Confirmation of the predicted binding sites was performed using Quantitative real-time PCR (Q-PCR). Detailed procedures are described previously [20].

### Quantitative reverse transcription-PCR (QRT-PCR)

QRT-PCR was performed as previously described [21]. Briefly, total RNA was isolated using a phenol-free and filter-based RNA isolation system (Qiagen) digested with DNase I to remove DNA contamination. Primer sequences for qRT-PCR were designed using Primer Express® software (Applied Biosystems, Foster City, CA) and are listed in Table 1.

**Table 1:** Primer Sequences on QRT-PCR.

ANPa F	CCC TGG AGG TGC TGG CTT TGG T
ANPa R	CCT CTC AAG GCT ACT GGG CTC A
TNNI1 F	CAA GGT GCT GTC TCA CTG CCA C
TNNI1 R	GCG CTC CTC GTG CTC CTG CTC CC
ACTA2 F	CTG TTC CAG CCA TCC TTC AT
ACTA2 R	TCA TGA TGC TGT TGT AGG TGG
BSP II F	GCC ACT CAC TGC CTT GAG CCT GC
BSP II R	CAT TGA GAA AGC ACA GGC CAT TC
NF-H F	CCG ACA TTG CCT CCT ACC
NF-H R	GGC CAT CTC CCA CTT GGT
LHX3 F	CAG CGT TCA GGA GGG GCA GGA C
LHX3 R	CTC CAT GCT CCA GGG AGA AGT TG
MAP2 F	CTC TCC TGT GTT AAG CGG AAA
MAP2 R	AAT ACA CTG GGA GCC AGA GC
SPARC F	AGT AGG AGA ATT GAT GAT GGT G
SPARC R	CAT CCA GCT CGC ACA CCT TGC CG
HES1 F	GCT GGA GAA GGC GGA CAT TCT GG
HES1 F	CTC GCA CGT GGA CAG GAA GCG GG
COL4A1 F	CAA AAG GAT CTG TTG GTG GAA TGG
COL4A1 R	GGG CCA GGG ATG CCA GGC ACA CC
LTA F	GGG CTT CGT GCT TTG GAC TA
LTA R	GTG TCA TGG GGA GAA CCA A
FGF5 F	CTG GAG TGC AGT GGC ATG AT
FGF5 R	AGG CAG GAG AAT CCC TTA AAC C
GAPDHF	GAA GGT GAA GGT CGG AGT C
GAPDHR	GAA GAT GGT GAT GGG ATT TC
ABL1 F	CAG AGA AGG TCT ATG AAC TCA TGC
ABL1 R	GGT GGA TTT CAG CAA AGG AG

### SALL4 promoter constructs and Promoter assays

The 5′-flanking region of SALL 4 was amplified with primers (5′ primer: GGTAC- GCGTAATAGGGCCAACCTCCATGGGAAG; 3′ primer: GCAAAGCTTCGACATGG- TGCGAGCATCGG) to generate a fragment from nucleolide (Nt) -1 to Nt-2102 upstream of the start codon ATG with MluI and HindIII sites at each end respectively. Genomic DNA isolated from human HEK293 cells was used as a template. The amplified PCR (polymerase chain reaction) fragment was cloned into the promoter-less pGL3-basic luciferase reporter plasmid (Promega, Madison, WI) to generate a SALL4 plasmid (P2102). The human OCT4 promoter reporter plasmid (Nt-1 to −1500), mouse Sall4 promoter fusion reporter plasmids containing fragments from Nt-1 to −2200, −645, −250, −190 and −l50 were created in the same manner as P2102.

Promoter luciferase assays were performed with the Dual-Luciferase Reporter Assay System (Promega, Madison WI), Twenty-four hours after transfection, cells were extracted with the use of a passive lysis buffer; a 20-µl aliquot was used for luminescence measurements with a luminometer. The data are represented as the ratio of firefly to Renilla luciferase activity (Fluc/Rluc). These experiments were performed in duplicate.

### SALL4 knockdown and human ES cell differentiation

Knock down of endogenous Sall4 expression was conducted using the same method as we reported previously [23]. Briefly, four short-hairpin RNA-expressing plasmids, 2 control (pRS, pRS-gfp) and 2 SALL4 specific (#7410, #. 7412; all four plasmid were obtained from Origen, Rockville, MD), were transfected into Phoenix packaging cells (Orbigen, San Diego, CA) using Lipofectamine 2000 (Invitrogen, Frederick, MD). Shed virus was harvested 48 hours after transfection, and control or stable SALL4 knockdown H9 clones were obtained under puromycin (1.2 µg/mL) selection after 7 days. ES cell differentiation was monitored by morphology inspection under microscope as well as alkaline phosphatase staining as previously described[13].

### Gel electrophoresis and Western blot analysis

Sodium dodecyl sulfate–polyacrylamide gel electrophoresis (SDS-PAGE) was carried out in SDS 10% wt/vol polyacrylamide slab gels as previously described [21], and the proteins were then transferred to nitrocellulose membranes. Immunoblotting was performed with a SuperSignal West Pico detection system as described by the manufacturer (Thermo Scientific Life Inc, Rockford, IL).

## Results

### Spontaneous differentiation of SALL4-reduced ES cells

**Figure 1:**
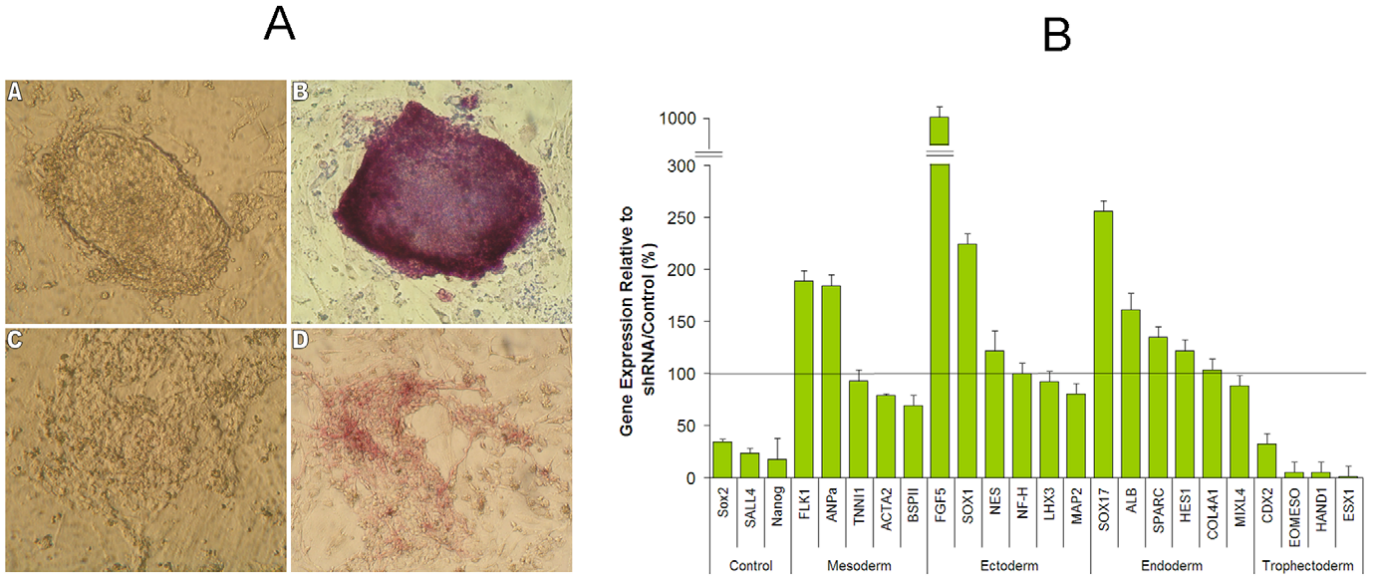
Differentiation of SALL4-reduced ES cells. (A) 24 hours after infection with either a control virus (A, B) or a SALL4-specific shRNA retrovirus (C, D), H9 ES cells were observed under light microscopy for morphological changes. The H9 ES cells treated with control viruses retain the ability to form ES colonies (A) and are positive for alkaline phosphatase (B), while the H9 ES cells treated with SALL4 shRNA viruses lose the colony-forming ability (C) and staining for alkaline phosphatase (D). (B) Lineage specific markers of ES cell lineages were evaluated for mRNA expression using QRT-PCR after infection with a SALL4- specific shRNA virus or a control virus. Mean values are plotted as a percentage relative to the control vector.

In murine ES cells, both over and under-expression of Sall4 causes differentiation [13]. However, little is known about the role of SALL4 in human ES cells. To this end, ES cells from the H9 human ES cell line were infected with either retroviruses expressing SALL4-specific short-hairpin RNAs (shRNA) or vectors viruses. Two SALL4 specific (#7410, #7412) and two scramble control (PRS, PRS-GFP) shRNAs were used for these experiments. SiRNA #7410 and #7412 have been used to successfully decrease Sall4 level in human leukemia NB4 cell line, and were able to consistently reduce SALL4 expression up to 75% when compared with the control in various cell line [23]. Here we applied these two shRNAs respectively to H9 cells, and down-regulation of endogenous SALL4 proteins were demonstrated by western blot (File S1). Following infection, morphological examination by light phase microscopy revealed distinct morphological changes in SALL4-reduced ES cells when compared with the control (Figure 1A). Similarly, alkaline phosphatase staining was significantly decreased in SALL4-reduced ES cells indicating a loss of pluripotency. Next, we sought to determine whether deceased SALL4 expression in H9 ES cells can lead to certain lineage differentiation. Using QRT-PCR we measured levels of mRNA for genes known to be markers of different cell lineages. Analysis revealed increased expression levels of genes associated with the endoderm, ectoderm, and mesoderm while markers for the trophectoderm layer showed no significant increase in expression levels (Figure 1B). This suggests that differentiation associated with reduced SALL4 expression causes differentiation toward endodermal, ectodermal, and mesodermal layers, and implicates SALL4 as a regulator of human ES cell fate. In addition, we observed that when SALL4 was down-regulated in mouse ES cells or human ES cells (File S1), the expression of Oct4 was decreased, as well as when SALL4 was over-expressed in human ES cells, the expression of Oct4 was up-regulated (File S1).

### A Novel SALL4/OCT4 transcription regulatory loop

#### Dose-dependent activation of human OCT4 promoter by SALL4 isoforms

**Figure 2:**
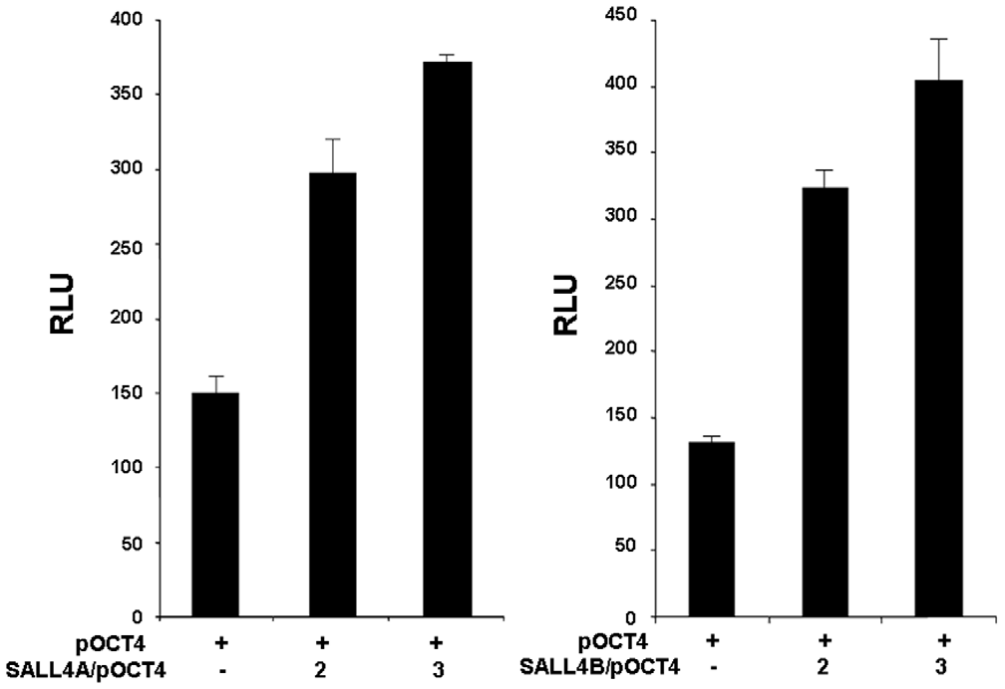
Dose-dependent activation of the OCT4 promoter by SALL4 isoforms. 0.3 µg of the OCT4-Luc promoter construct was co-transfected with 0.07 µg of Renilla plasmid and increasing ratios of either the SALL4A (left) or SALL4B (right) expressing construct into HEK-293 cells, pcDNA3 was used as the control. Y axis: relative luciferase unit (RLU). Data represent three independent experiments. Error bars denote standard deviation (SD).

We have identified that SALL4 has two isoforms [21]. It has been reported that Sall4 is able to bind and up-regulate Oct4 in murine ES cells [13]. However, it remains unclear which, if any, human SALL4 isoforms can activate human OCT4 transcription. To examine the correlation between the OCT4 promoter activity and expression of SALL4 isoforms, we first generated the human OCT4-Luc promoter reporter plasmid. An approximate 1.5-kb sequence upstream of the translation start site of OCT4 was subcloned into the 5′ upstream of the promoterless pGL3-basic luciferase reporter vector. The SALL4 responsiveness of the OCT4 promoter was evaluated through co-transfection of 0.3 µg of the OCT4 promoter construct and 0.07 µg of the Renilla plasmid together with increasing ratios of the SALL4A or SALL4B expression construct to the OCT4 promoter plasmid (ratios of 0 to 2). OCT4 promoter activity was increased more than 100% by co-transfection with an approximate 2-fold excess of the SALL4 constructs. When this experiment was repeated with the use of an increased molar excess of the SALL4A or SALL4B construct, the promoter was further activated to a significant degree (Figure 2). The expression of SALL4A or B by co-transfection was confirmed in File S1. These results are similar to previous reports on murine Oct4 activation by Sall4 [13], and confirm that both SALL4 isoforms are involved in activation of OCT4.

#### Characterization of the Transcriptional Activity of the SALL4 Promoter

**Figure 3:**
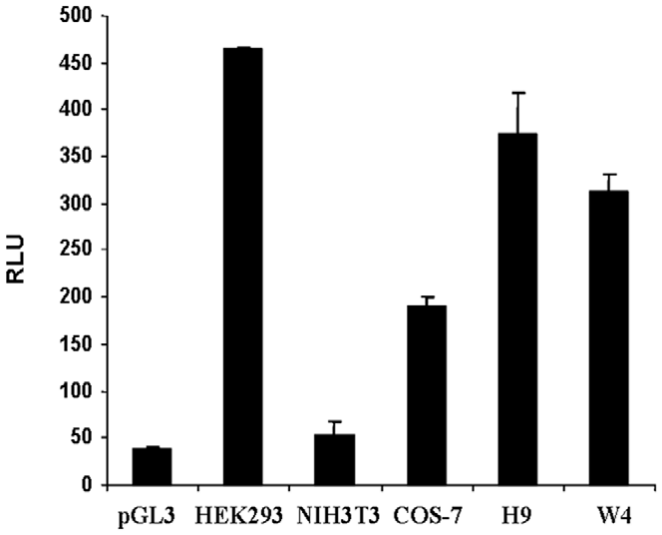
SALL4 promoter activities in HEK-293, NIH-3T3, COS-7, H9, and W4 Cells. The 5′-flanking region of SALL4 from −1 to −2102 upstream of the translation initiation codon (SALL4 P2102) was used to drive the expression of a luciferase reporter. Relative luciferase activity was calculated as arbitrary luciferase activity of SALL4 P2102 over that of the vector, pGL3-Basic, without the SALL4 promoter. Y axis: relative luciferase unit (RLU). Data represent three independent experiments. Error bars denote standard deviation (SD).

Sequencing of the 5′-flanking region of SALL4 did not reveal a classic TATA or CAAT box. This region, however, was GC-rich and contained several GC boxes. To determine SALL4 promoter activity, a 2.1-kb segment upstream of the ATG translation site was subcloned into the pGL3-basic vector, Luciferase activity driven by the SALL4 promoter in transient transfection assays was tested in various cell types including HEK-293, NIH-3T3 fibroblasts, COS-7, H9 human ES cell and W4 mouse ES cells. As expected, SALL4 promoter activity was higher in stem cell and embryonic kidney cell lines but lower in fibroblasts, as reflected by luciferase assays. The luciferase activity in the tested cells was, respectively, 12, 1.4, 5, 9.8 and 8.2- fold higher than the promoter-less pGL3 vector. The highest level of reporter gene was detected in HEK293 cells (Figure 3).

#### OCT4 dramatically activates the SALL4 promoter activity and this activation is inhibited by over-expression of SALL4A and SALL4B

**Figure 4:**
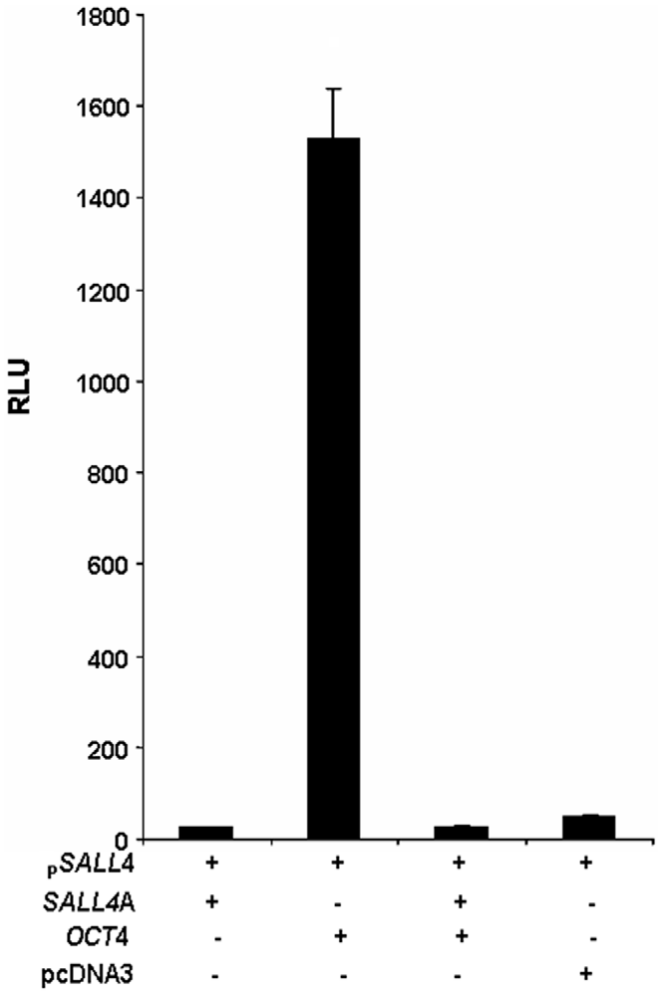
Promoter activity of SALL4 is dramatically enhanced by over-expressing OCT4, and SALL4 negatively regulates itself. 0.3 µg of SALL4 P2102 was cotransfected with 0.07 µg of the Renilla reporter and 0.9 µg of either a SALL4A expression plasmid (first column), or an OCT4 expression plasmid (second column). The third column represents cotransfection of both the SALL4A and OCT4 expression plasmids. Relative luciferase activity is depicted relative to the activity of SALL4 cotransfected with the pcDNA3 control vector (fourth column). Y axis: relative luciferase unit (RLU). Data represent three independent experiments. Error bars denote standard deviation (SD).

It was reported that murine Oct4 can bind to a murine Sall4 promoter region in a genomic ChIP-chip study [24], though it has not been verified. As many key transcription factors co-regulate each other in ES cells, we sought to confirm whether human OCT4 regulates SALL4 promoter activity. To determine this, 0.5 µg of the SALL4 reporter construct P2102 was cotransfected with 0.07 µg of the Renilla plasmid and 1.0 µg of the OCT4 expression construct (kindly provided by Dr. Peter Gruss, Max Planck Society, Germany). In HEK293 cells, expression of OCT4 strikingly up-regulates the SALL4 promoter activity over 50- fold (Figure 4) than by the pcDNA control. A similar stimulating effect by Oct4 was also observed in mouse Sall4 promoter assay (data not shown). Interestingly however, extra co-transfection with same amount of full-length SALL4A expression construct reversed this activation and the overall SALL4 promoter activity dropped to basal levels, suggesting that SALL4 may negatively regulates itself. The protein expression of the co-transfection was confirmed in File S1.

#### Self-repression of SALL4 in ES cells

**Figure 5:**
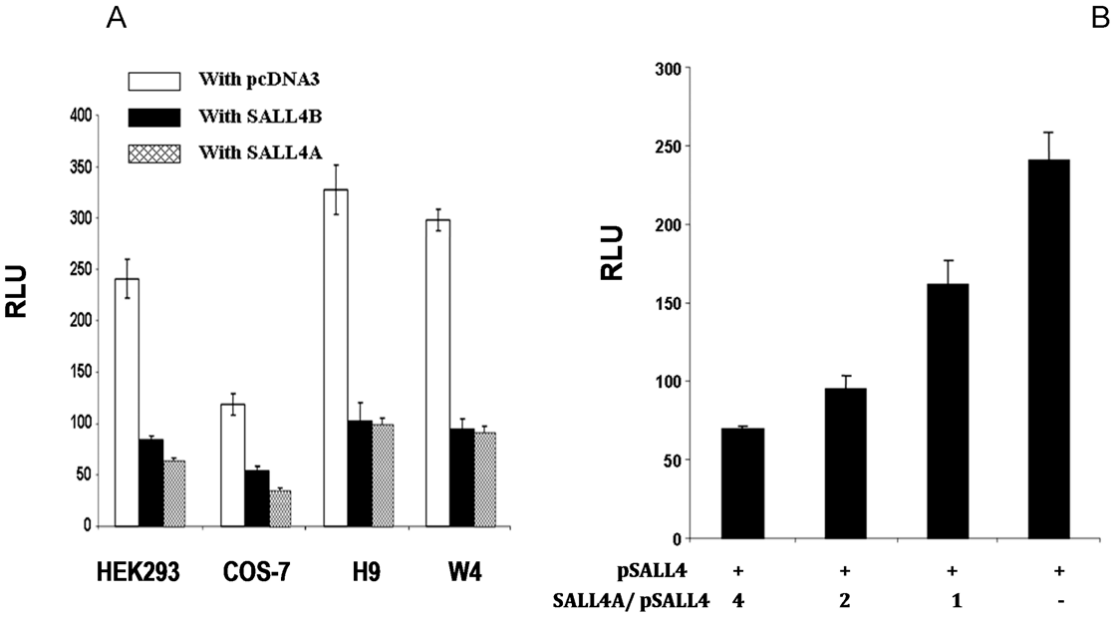
Auto-negative regulation of SALL4 isoforms. (**A**) *Self-suppression of the SALL4 promoter by SALL4A and SALL4B in different cell types*. 0.3 µg of SALL4 P2102 was cotransfected with 0.07 µg of the Renilla reporter and 0.9 µg of either SALL4A (hatched bars) or SALL4B (black bars) expressing plasmid in four different cell lines (HEK-293, COS-7, human ESC H9 and mouse ESC W4). The pcDNA3 empty vector was used as control (white bars), and the luciferase activity was normalized to Renilla reporter activity. Y axis: relative luciferase unit (RLU). Data represent three independent experiments. Error bars denote standard deviation (SD). (**B**) *SALL4 suppresses its own promoter activity in a dose- dependent manner in human embryonic stem cells.* Using an approach similar to Figure 3, in human ES H9 cells, 0.3 µg of SALL4 P2102 was cotransfected with 0.07 µg of the Renilla reporter and increasing ratios of SALL4A. First bar, 4∶1; second bar, 2∶1; third bar, 1∶1; fourth bar, pcDNA3 control. When the ratio of SALL4A to SALL4 promoter reporter reached 4∶1, the promoter activity dropped approximately 3 fold when compared with the basic level. Y axis: relative luciferase unit (RLU). Data represent three independent experiments. Error bars denote standard deviation (SD).

To confirm the above mentioned SALL4 promoter auto-regulation, the human SALL4 P2102 construct was co-transfected with either SALL4A or SALL4B expression plasmids in HEK-293, COS-7, H9 and W4 cells. Twenty four hour-post transfection, the self-suppression of SALL4 (both A and B isoforms) on its own promoter activity was observed in all cell lines tested (Figure 5A). Further, this self-suppression is dose dependent. In human ESC H9 cells, when the ratio of SALL4A with SALL4 promoter reached 4∶1, the promoter activity dropped approximately 3.5 fold compared with the basal level (Figure 5B). Similar dose dependent results were also observed in SALL4B (data not shown). We then decided to further our studies by mapping the SALL4 auto-regulation site. The protein expression of the co-transfection was confirmed in File S1.

#### Identification of SALL4 self-regulation binding site

**Figure 6:**
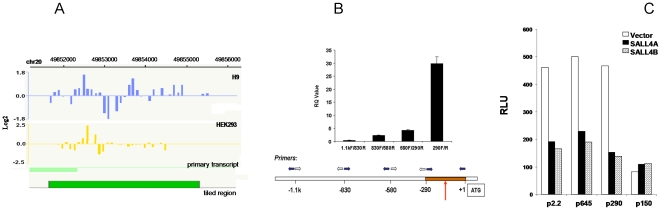
Identification of SALL4 self-regulation binding site. (**A**) *Human SALL4 binds to its own promoter region detected by ChIP-chip in human ES H9 and HEK 293 cells.* ChIP assays were performed by using an antibody against SALL4. Enriched chromatin was extracted and analyzed by NimbleGen standard procedures. A high signal peak representing significant binding of chromatin, which resides within 500 bp area above the transcription start site, can be observed in both cell types. (**B**) *SALL4 binding site on its own promoter confirmed by quantitative PCR (QPCR).* Enriched chromatin by ChIP assays was analyzed by QPCR with primers as indicated by the arrows. ChIP assays were performed using SALL4 antibody in H9 ES cells to detect the endogenous SALL4 binding site. Relative enrichment of SALL4 promoter regions (amplicons) were quantitated by QPCR. Pull-down input was used as a control. SALL4 appears to bind to its own promoter within the −290 to +1 bp relative to its translation start (ATG) site. Y axis: relative real-time PCR value when compared to input (RQ Value). Data represent three independent experiments. Error bars denote standard deviation (SD). (**C**) *Mapping of the SALL4 repressive functional site within its own promoter region by a luciferase reporter assay.* In HEK-293 cells, 0.3 µg of different length SALL4 promoter constructs were co-transfectecd with 0.07 µg of the Renilla luciferase plasmid and 0.9 µg of either SALL4A or SALL4B plasmid. While the basal luciferase activity decreased as promoter length decreased from −290 (P290) to −150 (P150), the region between −250 to −190 seemed to be the self-repression functional site since there was no repression effect observed in P190 and P150. Y axis: relative luciferase unit (RLU). Data represent the mean of three independent experiments.

Using an anti-SALL4 antibody, we first performed ChIP-chip assay, and identified SALL4 binding sites on its own promoter region in both 293 cells and H9 ES cells (Figure 6A).

To further confirm this finding, primers that covered the SALL4 promoter region were designed and used to map the SALL4 binding sites more precisely. The forward and reverse primer set (1–2) amplified strong 290-bp amplicons from the immunoprecipitates in H9 ES cells (Figure 6B). Immunoprecipitation reactions using preimmuno serum show very little amplification of the SALL4 promoter in the immunoprecipitated DNA (data not shown). All ChIP samples were tested for false-positive PCR amplification by sequencing amplicon DNAs to ascertain the specificity of the SALL4 that bound to the cis-regulatory elements. The intensity of each PCR amplicon was also normalized against the ChIP input band to show the relative abundance of SALL4 that bound to its own promoter by quantitative real-time PCR (QRT-PCR) (Figure 6B). The observed binding was specific, as essentially no signal was observed in parallel ChIP experiments using cells transfected with an empty vector (pcDNA3).

To further investigate the putative SALL4 binding site on its own promoter, we next performed more SALL4 promoter assays. In this experiment, we generated a series of shortened SALL4 promoter constructs. Fragments from nucleotides −1 (upstream of the ATG translational site) to −2.2 kb, −645 bp, −290 bp and −150 bp (termed as p2.2, p645, p290 and p150) were PCR amplified respectively and subcloned into the pGL3-basic reporter vector. The SALL4 self repression assay was performed using each of the promoter constructs. As seen in Figure 6C, both SALL4 A and B isoforms suppress its own promoter activity in p2.2, p645 and p290 constructs: the promoter activities dropped by ∼4 fold when cells were cotransfected with either SALL4 A or B isoform. While the p150 promoter construct failed to show this self-suppression effect, suggesting the functional site of SALL4 on its own promoter is within the −290 region. This study further indicated that a region between −290 to +1 of the SALL4 promoter could be a binding site for SALL4.

### The SALL4/OCT4 network regulates other SALL4 gene family members and self- negative feedback is unique to SALL4

#### Repression of the promoters of SALL gene family members by SALL4 isoforms

**Figure 7:**
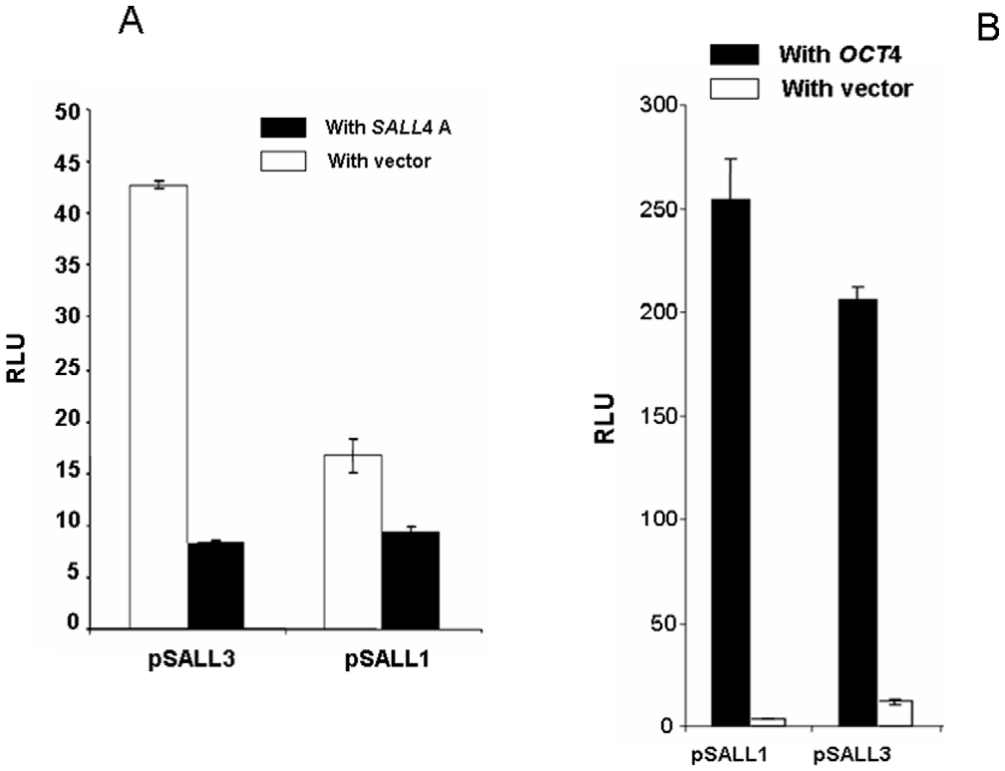
Regulations of SALL1 and SALL3 by Oct4 and *SALL4*. (**A**) *Repression of the promoters of SALL gene family members by SALL4 isoforms.* The effect(s) of *SALL4A* on other SALL gene member promoter activities were evaluated as following. 0.3 µg of the SAL-Luc promoter constructs (pSALL1, pSALL3) were co-transfected with 0.07 µg of Renilla luciferase plasmid and 0.9 µg of SALL4A expressing construct into HEK-293 cells; pcDNA3 was used as the control. Y axis: relative luciferase unit (RLU). Data represent three independent experiments. Error bars denote standard deviation (SD). (**B**) *Activation of the promoters of SALL gene family members by OCT4.* 0.3 µg of the SAL-Luc promoter constructs (pSALL1, pSALL3, pSALL4) were cotransfected with 0.07 µg of the Renilla luciferase plasmid and 0.9 µg of OCT4 expressing construct or pcDNA3 into HEK-293 cells. Twenty four hour post-transfection, luciferase activity was evaluated for each group. Expression of OCT4 strikingly stimulated the SALL promoter activities (15∼43 fold) when compare with that of pcDNA3 vector control, Y axis: relative luciferase unit (RLU). Data represent three independent experiments. Error bars denote standard deviation (SD).

To examine the effect of SALL4 isoforms on other SALL family genes, the SALL1 and SALL3 promoters were generated using the same pGL3-basic vector. An approximate 2.0-kb sequence upstream of the translation start site of SALL1 or SALL3 was subcloned into the 5′ upstream of the promoterless pGL3-basic luciferase reporter plasmid. The SALL4 responsiveness of the SALL1 or SALL3 promoter was evaluated through co-transfection with 0.3 µg of the SALL1 or SALL3 promoter construct and 0.07 µg of the Renilla plasmid together with 0.9 µg of the SALL4A expression construct. SALL1 or SALL3 or promoter activity was repressed for more than 2-fold or 3-fold respectively (Figure 7A). Similar results on repression of the promoters of SALL1 and SALL3 by SALL4B were observed as well (Data not shown). In addition, using an anti-SALL4 antibody, we performed chromatin immunoprecipitation followed by DNA microarray analysis (ChIP-chip), and identified the SALL4 binding sites on the promoter regions of SALL1 and SALL3 in 293 cells and H9 ES cells (Data not shown). The SALL4 binding sites were identical between the two different types of cells, indicating that using 293 cells for the promoter studies is equivalent to using H9 ES cells. These data suggest that SALL4 isoforms are able to regulate other members of the SALL gene family involving embryonic stem cell function.

#### Effect of OCT4 on other SALL gene member promoters

To determine if OCT4 stimulates the activity of other SALL gene member promoters, SALL1 and SALL3 promoter constructs (pSALL1 and pSALL3) were co-transfected with OCT4 in HEK-293 cells. As shown in Figure 7B, twenty four hours post-transfection, the overexpression of OCT4 strikingly stimulated the promoter activities of SALL1 and SALL3 when compared with that of the pcDNA3 vector control (15∼43 fold). The OCT4 binding sites on the promoter regions of SALL1 and SALL3 were reported previously[24].

**Figure 8:**
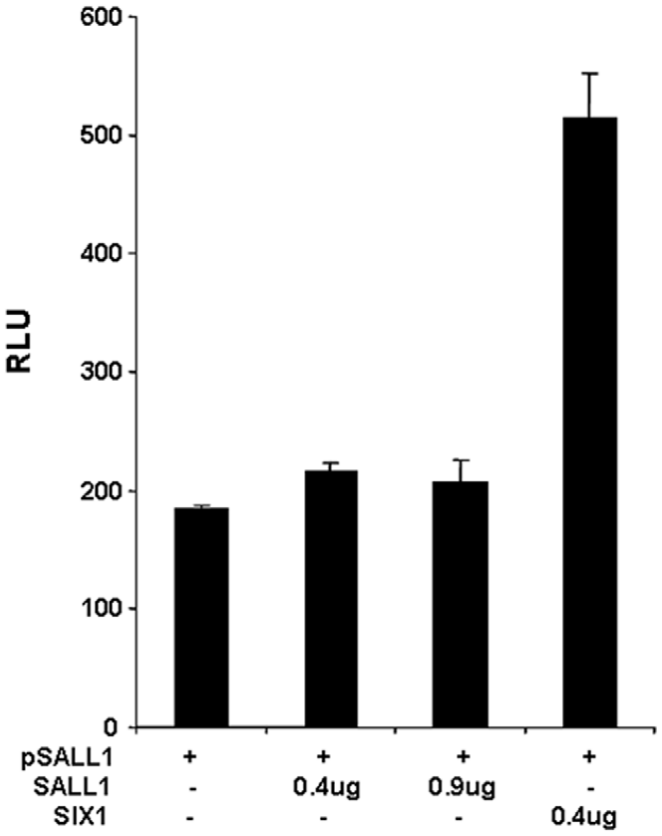
Negative self-regulation not present on other SALL family members. Using a similar approach in HEK293 cells, 0.3 µg of SALL1 -Luc (pSALL1) was cotransfected with 0.07 µg of Renilla reporter, and 0.9 µg of SALL1- pcDNA3, or 0.9 µg of pcDNA3, or 0.4 µg SALL1-pcDNA3 plus 0.4 µg SIX1-pcDNA3. Luciferase assay were performed 24 hr post transfection, and luciferase activities were normalized against Renillar reporter activity, Y axis: relative luciferase unit (RLU). Data represent three independent experiments. Error bars denote standard deviation (SD).

We also tested whether other SALL members had self-suppression autoregulation. We found that this observation was unique to SALL4 and not true for other SALL members, for example, SALL1 (Figure 8) failed to demonstrate self-suppression of its own promoter. By comparison, we used a known activator and showed that the over-expression of SIX1 protein can activate the SALL1 promoter activity by ∼3 fold (Figure 8) as we reported previously [25]. The protein expression of the co-transfection was confirmed in File S1.

#### SALL4 blocks the Oct4 mediated activation of the promoters of the SALL gene family

**Figure 9:**
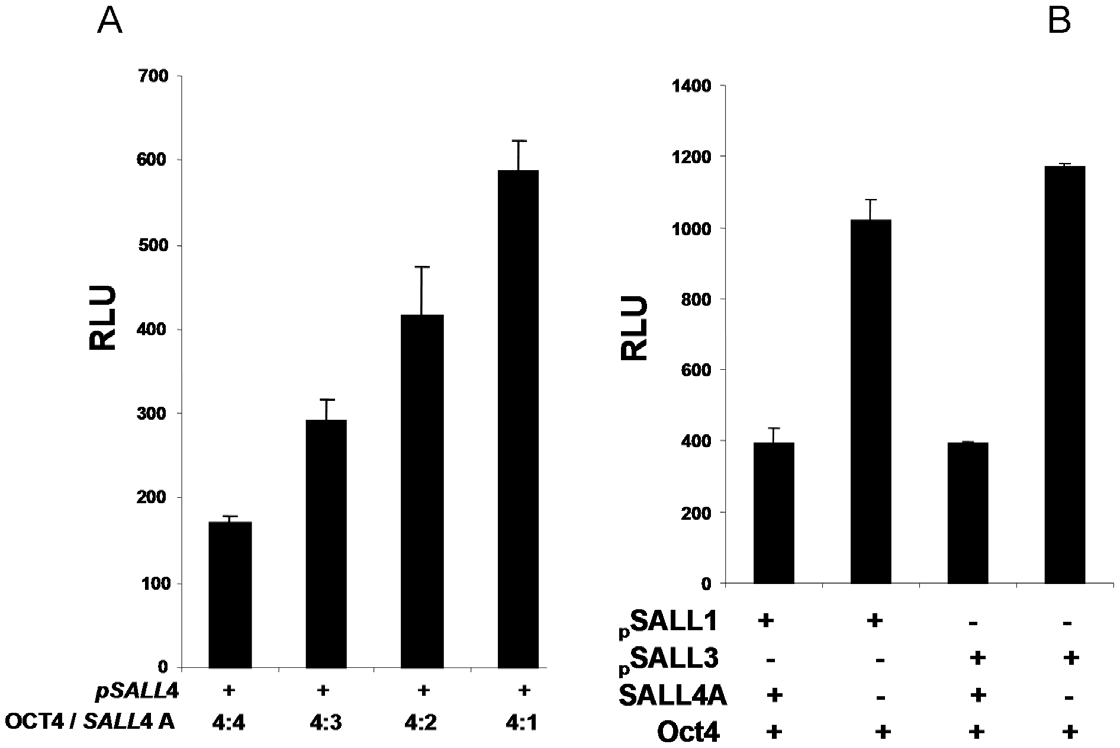
SALL4 blocks the OCT4-mediated activation of the promoters of members of the SALL gene family. (**A**) *SALL4 promoter activity depends on the expression level and ratio of SALL4 to OCT4.* HEK293 cells were transfected with 0.3 µg of SALL4-Luc reporter, together with different ratios of the SALL4A and OCT4 expression plasmids. Luciferase activities of cell lysate were analyzed 24 hr post transfection. Y axis: relative lucifierase unit (RLU). Data represent three independent experiments. Error bars denote standard deviation (SD). (**B**) *Activation of OCT4 on SALL1 and SALL3 promoters were altered by the presence of SALL4A.* HEK-293 cells in 24 well plate were transiently transfected with different SALL promoter reporters (pSALL1 or pSALL3; 0.3 µg), together with 0.6 µg of the OCT4 construct, and/or SALL4 A construct. pcDNA3 vector was used as control. Luciferase activities were normalized against Renillar reporter activity. Y axis: relative luciferase unit (RLU). Data represent three independent experiments. Error bars denote standard deviation (SD).

Since SALL4 and Oct4 have opposite effects on the promoters of the SALL gene family, we sought to investigate the combination effect of both factors on SALL gene promoters. By cotransfection of SALL4 promoter plasmid P2102, together with different amount of OCT4 and SALL4 expression constructs in HEK293 cells, we observed that the overall regulating effect of these two transcription factors on SALL4 promoter activity is strictly controlled by their expression level and ratios (Figure 9A). SALL4 expression is tightly regulated by self-repression and a positive feedback from OCT4. In addition, the activation of OCT4 on other SALL member promoters can be blocked by the over-expression of SALL4 protein (Figure 9B).

## Discussion

SALL4 was initially identified as a homologue of Drosophila gene spalt. Its mutations lead to a range of congenial human developmental abnormalities including “DRRS” and IVIC. These findings suggest that SALL4 plays an important role in human developments. More recently, SALL4 was identified as a “stemness” factor involved in murine ES cells. Murine Sall4 is essential for inner cell mass formation, and knocking down Sall4 in murine ES cells leads to loss of pluripotency [13]. In this study, we are the first to show that SALL4 is required for the maintenance of human ES cell property.

The molecular mechanism(s) of SALL4 in maintaining the stem cell properties involve at least two processes: SALL4 activates Oct4 as a transcriptional factor, and interacts with Nanog by forming a protein-protein complex. It seems that SALL4 is a “core factor” for this SALL4/Nanog/Oct4 network [26], [20]. In a murine ES cell genome-wide ChIP-chip analysis, we have shown that Sall4 bound twice as many annotated genes within promoter regions as Nanog and four times as many as Oct4 [20]. In addition, SALL4 has been implicated in recruiting the epigenetic repressor complex, Mi-2/Nucleosome Remodeling and Deacetylase (NuRD), in ES cells[27].

This leads to a very intriguing question: how is SALL4 regulated? We have previously reported that human SALL4 has two isoforms, which prompts us to study whether these two isoforms have differential effects. In this study, we have shown that both SALL4 isoforms can activate OCT4, and are positively regulated by OCT4. Since SALL4 and OCT4 form a positive feedback loop, there must be some type of negative regulator mechanism present to balance the proper expression(s) of SALL4 and OCT4. We have discovered that SALL4 poses a strong self-repressive auto-regulation, which in turn, acts as a “gate keeper “or a “break” for the SALL4/OCT4positive feedback loop.

Other SALL gene family members, in particular, SALL1 and SALL3, have been shown to be present at very early embryonic developmental stages both in human and mice. Heterozygous mutations of SALL1 are associated with TBS, a congenital malformation that includes deformations in digit, heart, ear, kidney and limbs. Sall1 null mice die soon after birth due to renal agenesis. A “knock-in” mouse model resembles human TBS more closely, indicating that domain-negative effort is responsible for the pathogenesis of TBS. There is a significant overlap in the phenotypes of DRRS and TBS. Interestingly, the Sall1 mutant can bind and potentially interact with SALL4, and mutant SALL4 has been shown to form a complex with Sall1 as well [28]. These probably account, at least in part, for the overlapping of the features between DRRS and TBS. SALL3 has been implicated in 18q- syndrome with multiple organ defects. Sall3 null mice die soon after birth due to defects in neural plate developments [5].

Given the essential roles that the SALL gene family members play during development, it is of great interest to explore the interactions between the SALL gene family members, as well as the mechanism(s) which regulate their expressions. In this study, we have shown that consistent with its self-repression function, SALL4 represses the activities of the promoters of SALL1 and SALL3. This is antagonized by the activation of OCT4 on the promoters of SALL1 and SALL3.

**Figure 10:**
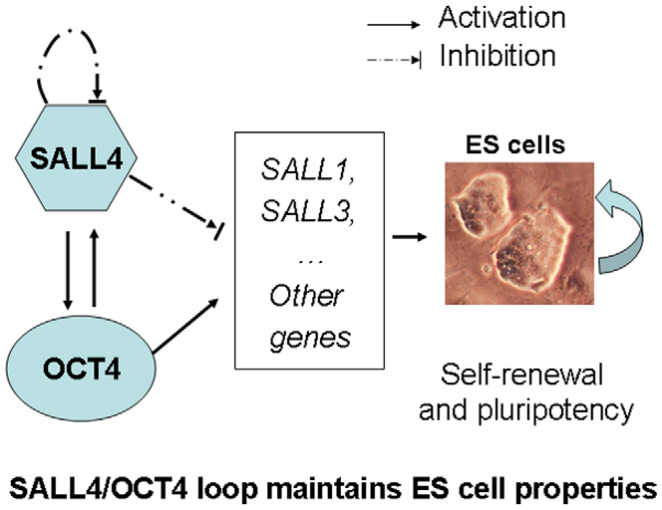
Hypothesis on the SALL4/OCT4 transcriptional feedback loop in ES cells. SALL4 and OCT4 form a regulatory feedback network whereby SALL4 isoforms activate OCT4; in return, OCT4 activates SALL4 isoforms as well, As a “break” for this positive feedback loop, SALL4 possesses a strong self-repress effect, which, seems to set a tight regulation for the proper expressions of both genes. This regulatory network affects the expressions of other SALL gene family members, such as SALL1 and SALL3.

Based on the above findings, we propose the following hypothesis (Figure 10): SALL4 and OCT4 form a regulatory feedback network whereby SALL4 isoforms activate OCT4; in return, OCT4 activates SALL4 isoforms as well. As a “break” for this positive feedback loop, SALL4 possesses a strong self-repressive effect, which, seems to set a tight regulation for the proper expression of both genes. While the detailed mechanism on how SALL4 represses itself still remains unknown, we have shown that SALL4 can recruit the NuRD deacylacation complex. The NuRD epigenetic repressor complex has been mainly associated with gene repression, and we have shown that SALL4 can inhibit the transcription of PTEN and SALL1 through NuRD [27]. It is possible that the self-repression of SALL4 is mediated, at least in part, through this epigenetic repressor complex. In addition, since this model is based on studies using co-transfections of promoter constructs, additional future studies such studies of the biochemistry of DNA binding will be necessary to refine and confirm it.

In summary, SALL4 appears to play a dominant role in the SALL4/OCT4 regulatory network. This transcriptional network seems to regulate other SALL gene family members as well, such as SALL1 and SALL3. In addition to its essential role in ES cells, SALL4 is found to be involved in adult tissue stem cells and leukemic stem cells. It is worthy to point out that SALL4 is one of the few genes, if not the only one, that is involved in stem cell properties shared by ES and adult tissue stem cells. More in-depth studies on SALL4 should add to our understanding of the “stemness” feature shared by all stem cells.

## Supporting Information

File S1 Supplemental figures. Figure S1. Down-regulation of SALL4 protein in human ES cells. The human ES H9 cells were infected with retroviruses either expressing two SALL4-specific shRNAs (#7410 and #7412) or scramble control (PRS, PRS-GFP) shRNAs. Whole cell lysates were immunoblotted with anti-SALL4 as described in [21] or anti β-actin antibodies (Abcam) to ensure equal loading of proteins. Figure S2. Expression of OCT4 is affected by SALL4 in the ES cells. (A) Down-regulation of SALL4 in murine ES cells led to decreased expression of Oct4. Following adenovirus induced removal of one of Sall4 alleles in Sall4flox/+ ES cells, expression of Oct4 is decreased as measured by Q-RT-PCR. The Sall4/Gapdh ratio in control cells was set at 1. The values are the mean of triplicate. (B) Down-regulation of SALL4 in human ES cells led to decreased expression of OCT4. OCT4 protein level was decreased by down-regulation of SALL4 in H9 ES cells through infecting cells with retroviruses expressing SALL4-specific shRNAs (#7410 or #7412) when compared to those infected with scramble control (PRS, PRS-GFP) shRNAs. Whole cell lysates were immunoblotted with anti-OCT4 (Santa Cruz Biotechnology, Inc) or anti β-actin antibodies (Abcam). (C) Overexpression of SALL4 A or B isoform in H9 ES cells resulted in increased OCT4 protein expression. Membranes were probed with anti-OCT4 or anti β-actin antibodies as described as above. Figure S3 Overexpression of SALL4 isoforms in HEK-293 cells. Either the SALL4B-HA (lane 1, 2, 3) or SALL4A-HA (lane 4, 5, 6) expressing construct was transfected into HEK-293 cells, with different amount (0.5, 1.0 1.5 mg). Western membrane was probed with anti-HA (Bethyl Laboratories, Inc) or anti β-actin antibodies (Abcam). Figure S4. Western blot analysis of total cell extracts from HEK293 cells cotransfected with OCT4 and SALL4-HA expressing vectors (lane 1) or transfected either SALL4-HA alone (lane 2) or pcDNA3 vector (lane3). Membrane was probed with anti-HA or anti OCT4 antibodies as described above. Figure S5. Over-expression of SALL4 isoforms in COS7, mouse W4 ES and human H9 ES cells. Western blot analysis of total cell extracts from either Cos-7 cells (A), W4 ES cells (B), or H9 (C) which were transfected with either SALL4A-HA, SALL4B-HA or pcDNA3 vector. Membrane was probed with anti-SALL4 antibodies as described above. Figure S6. Expression of SIX1 and SALL1 in HEK293 cells SALL1-6xhis or SIX1-6xhis expression construct were transfected into HEK293 cells in this study, western blot analysis of total cell extracts 48 hours after transfection were performed using anti-his antibody. Left lane was extracts from pcDNA3 vector control. Membrane was probed with anti-his antibodies (Bethyl Laboratories, Inc).
(0.11 MB PDF)
