# Supplementary figures and images for: A Novel SALL4/OCT4 Transcriptional Feedback Network for Pluripotency of Embryonic Stem Cells
Source: PLoS One. 2010 May 21;5(5):e10766. doi: 10.1371/journal.pone.0010766 (PMC2874005; doi:10.1371/journal.pone.0010766)

Figure S1

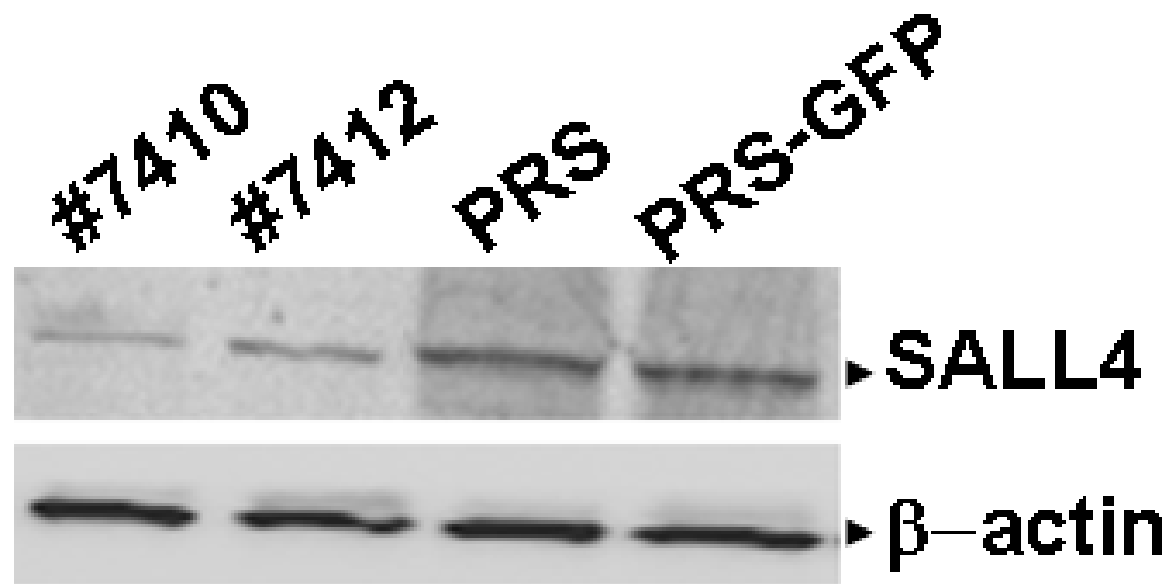

Figure S2

A

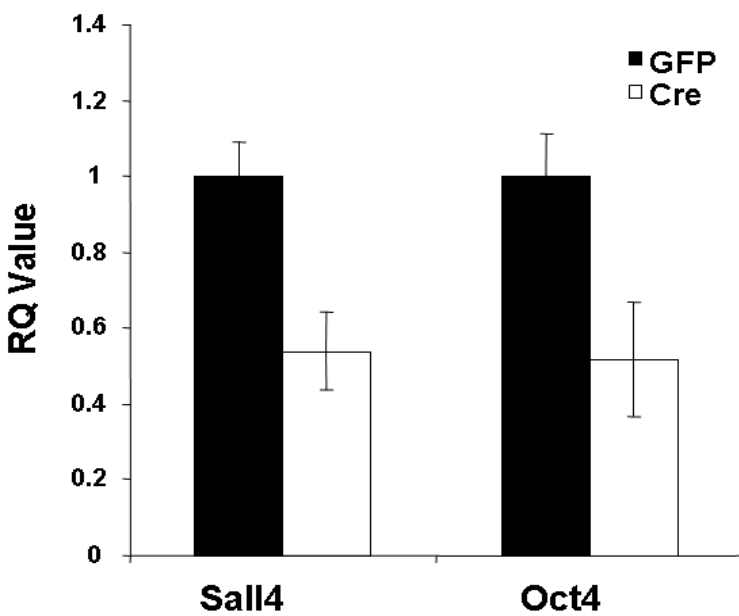

B

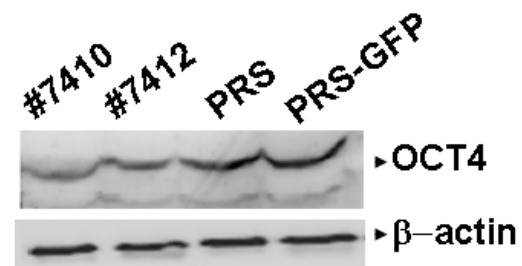

C

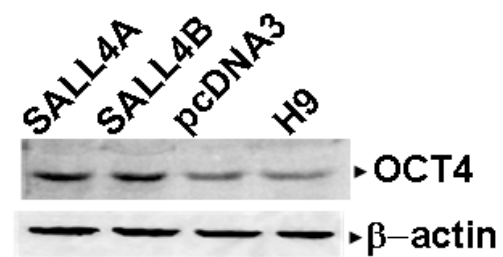

Figure S3

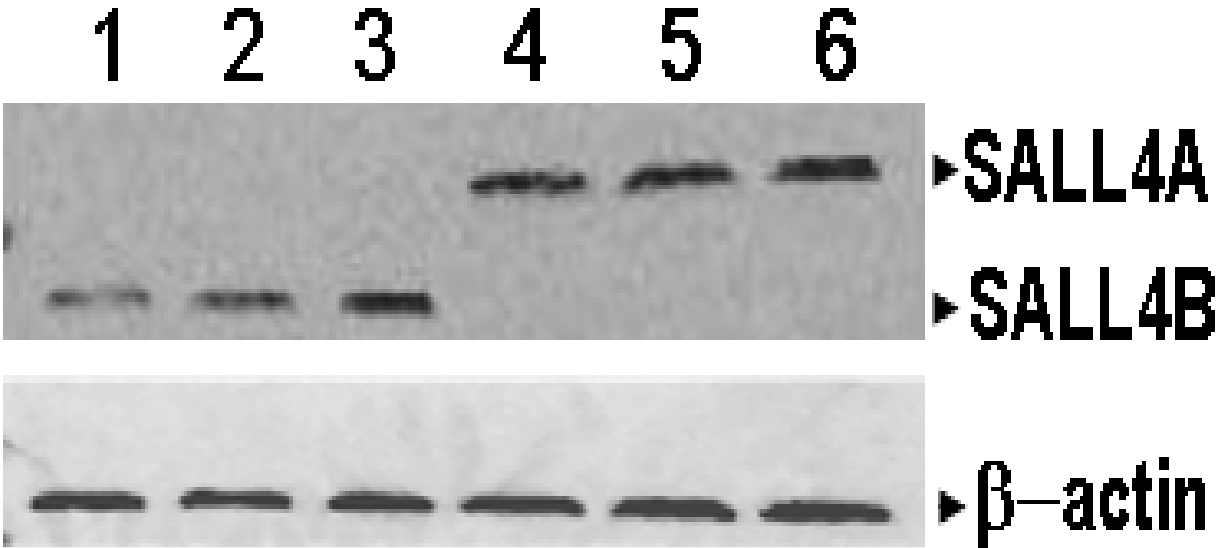

Figure S4

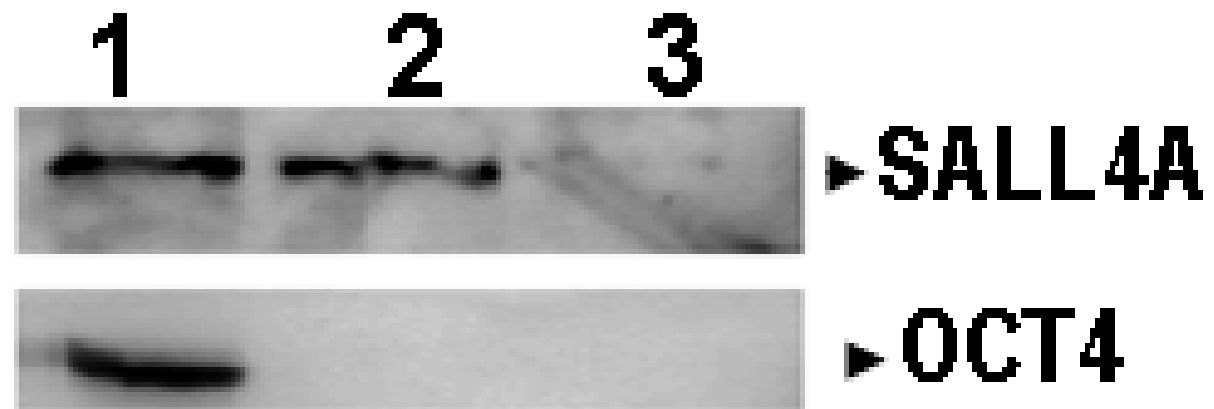

Figure S5

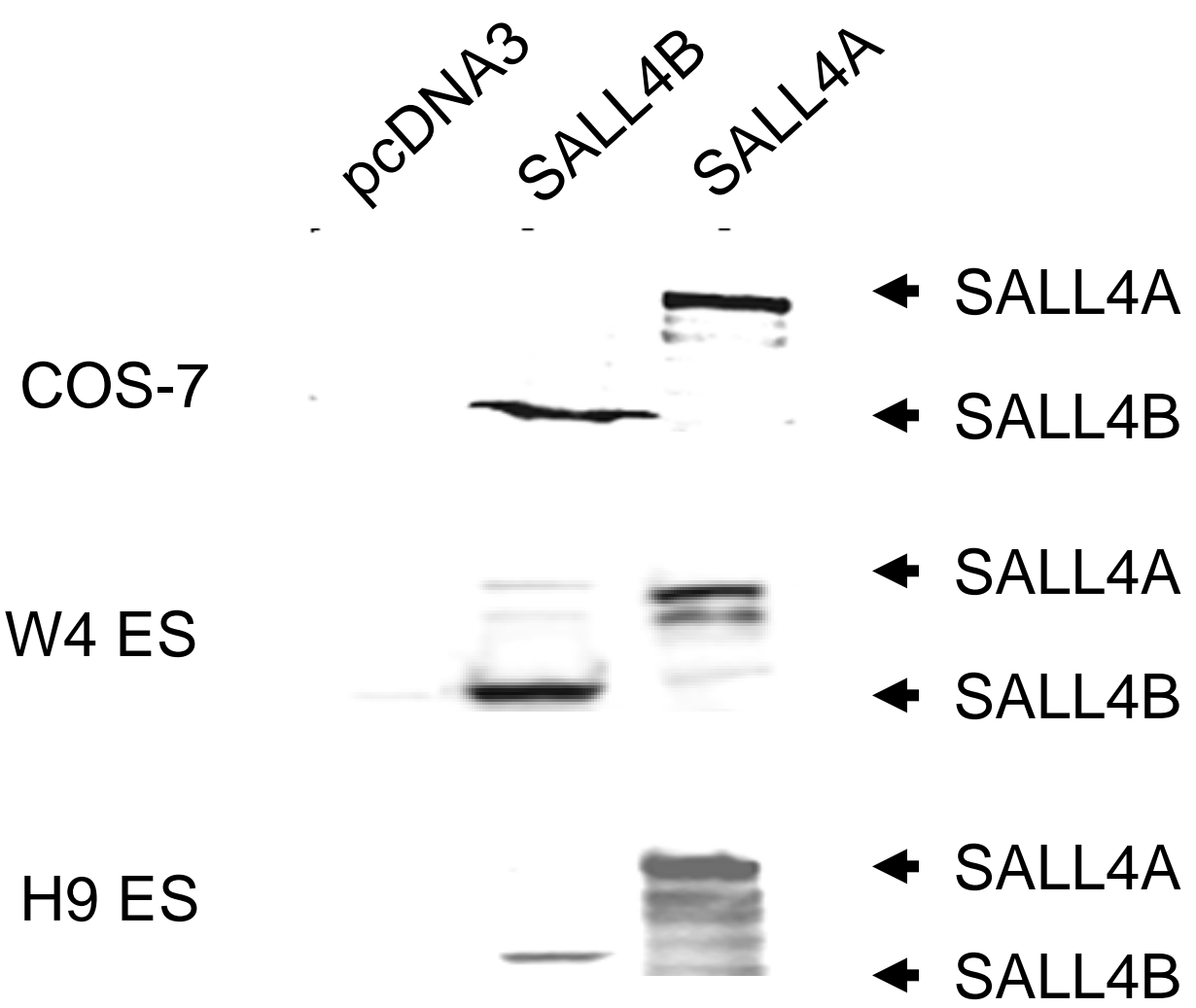

Figure S6

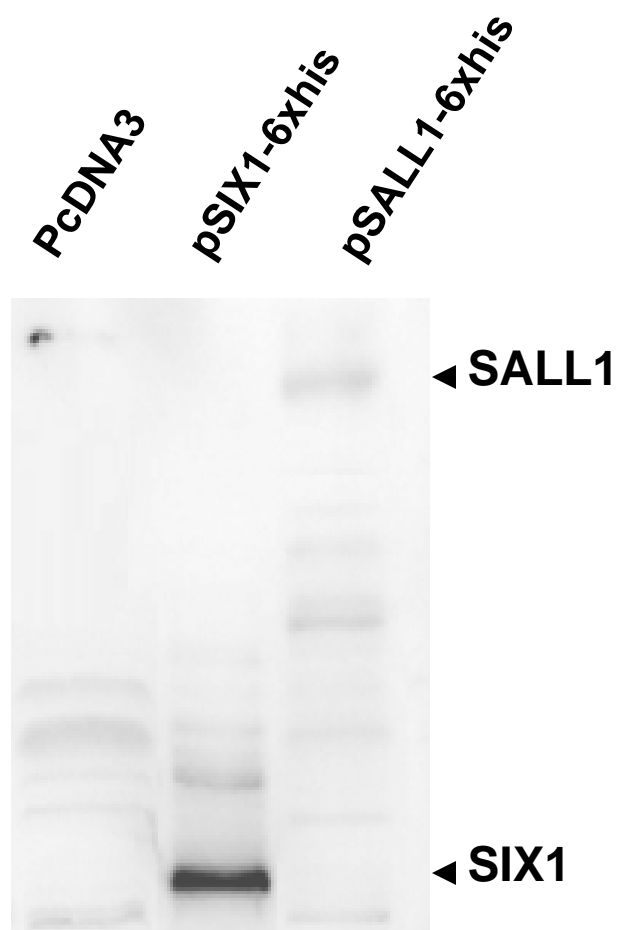

Supplement: File S1 — Supplemental figures. Figure S1. Down-regulation of SALL4 protein in human ES cells. The human ES H9 cells were infected with retroviruses either expressing two SALL4-specific shRNAs (#7410 and #7412) or scramble control (PRS, PRS-GFP) shRNAs. Whole cell lysates were immunoblotted with anti-SALL4 as described in [21] or anti β-actin antibodies (Abcam) to ensure equal loading of proteins. Figure S2. Expression of OCT4 is affected by SALL4 in the ES cells. (A) Down-regulation of SALL4 in murine ES cells led to decreased expression of Oct4. Following adenovirus induced removal of one of Sall4 alleles in Sall4flox/+ ES cells, expression of Oct4 is decreased as measured by Q-RT-PCR. The Sall4/Gapdh ratio in control cells was set at 1. The values are the mean of triplicate. (B) Down-regulation of SALL4 in human ES cells led to decreased expression of OCT4. OCT4 protein level was decreased by down-regulation of SALL4 in H9 ES cells through infecting cells with retroviruses expressing SALL4-specific shRNAs (#7410 or #7412) when compared to those infected with scramble control (PRS, PRS-GFP) shRNAs. Whole cell lysates were immunoblotted with anti-OCT4 (Santa Cruz Biotechnology, Inc) or anti β-actin antibodies (Abcam). (C) Overexpression of SALL4 A or B isoform in H9 ES cells resulted in increased OCT4 protein expression. Membranes were probed with anti-OCT4 or anti β-actin antibodies as described as above. Figure S3 Overexpression of SALL4 isoforms in HEK-293 cells. Either the SALL4B-HA (lane 1, 2, 3) or SALL4A-HA (lane 4, 5, 6) expressing construct was transfected into HEK-293 cells, with different amount (0.5, 1.0 1.5 mg). Western membrane was probed with anti-HA (Bethyl Laboratories, Inc) or anti β-actin antibodies (Abcam). Figure S4. Western blot analysis of total cell extracts from HEK293 cells cotransfected with OCT4 and SALL4-HA expressing vectors (lane 1) or transfected either SALL4-HA alone (lane 2) or pcDNA3 vector (lane3). Membrane was probed with anti-HA or anti [file pone.0010766.s001.pdf]
